# Congo Red Decolorization Using Textile Filters and Laccase-Based Nanocomposites in Continuous Flow Bioreactors

**DOI:** 10.3390/nano10061227

**Published:** 2020-06-24

**Authors:** Natalia Lopez-Barbosa, Sergio Leonardo Florez, Juan C. Cruz, Nancy Ornelas-Soto, Johann F. Osma

**Affiliations:** 1 CMUA, Department of Electrical and Electronics Engineering, Universidad de los Andes, Cra. 1E No. 19a-40, Bogotá DC 111711, Colombia; n.lopez10@uniandes.edu.co (N.L.-B.); sl.florez10@uniandes.edu.co (S.L.F.); 2 Department of Biomedical Engineering, Universidad de los Andes, Cra. 1E No. 19a-40, Bogotá DC 111711, Colombia; jc.cruz@uniandes.edu.co; 3 Laboratorio de Nanotecnología Ambiental, Escuela de Ingeniería y Ciencias, Tecnológico de Monterrey, N. L., Monterrey 64849, Mexico; ornel@tec.mx

**Keywords:** azo dyes, decolorization, laccase, nanocomposites, *P. Sanguineus* CS43

## Abstract

Removal of azo and diazo dye content from textile industry wastewaters is crucial due to their environmental impact. Here, we report on the use of the fungal laccase from *Pycnoporus sanguineus* CS43 immobilized on silica nanoparticles and entrapped in textile-based filters for the degradation of Congo Red. Laccase immobilization and synthesis of the nanocomposites were carried out by two different methods, one in the presence of acetone and the second using water as solvent. This led to a change in the hydrophobicity of the obtained biofilters. Successful preparation of the nanocomposites was confirmed via FTIR spectroscopy. Changes in the secondary structure of the enzyme were inspected through the second derivative of the FTIR spectra. Six different types of filter were fabricated and tested in a continuous flow bioreactor in terms of their decolorization capabilities of Congo Red. The results indicate removal efficiencies that approached 40% for enzymes immobilized on the more hydrophobic supports. Backscattered electron (BSE) images of the different filters were obtained before and after the decolorization process. Percentage of decolorization and activity loss were determined as a function of time until a plateau in decolorization activity was reached. Experimental data was used to recreate the decolorization process in COMSOL Multiphysics^®^ (Stockholm, Sweden). These simulations were used to determine the proper combination of parameters to maximize decolorization. Our findings suggest that the treatment of textile-based filters with immobilized laccase in conjunction with hydrophobic nanocomposites provides a suitable avenue to achieve more efficient laccase dye decolorization (39%) than that obtained with similar filters treated only with free laccase (8%). Filters treated with silica-based nanocomposites and immobilized laccases showed an increase in their decolorization capability, probably due to changes in their wetting phenomena.

## 1. Introduction

Remediation of water effluents from the textile industry is imperative to counter their environmental impact as a consequence of their high contents of a number of toxic and recalcitrant compounds [1,2,3]. In this regard, some of the environmentally worrisome compounds include dyes, naphthol, nitrates, acetic acid, soaps, chromium compounds, and heavy metals, like copper, arsenic, lead, cadmium, mercury, nickel, and cobalt, which ultimately make these effluents highly toxic [4,5]. Of particular concern is the high content of salts and azo and diazo dyes, which have been reported to produce mutagenic reactions in organisms present in soil and water ecosystems [2,3]. About 70% of dyes present in textile effluents contain azo (-N=N-) chromophores [6], which are thought to be responsible for fluctuations in chemical oxygen demand, color, salinity and pH [3]. In addition, only 47% of the dyes are biodegradable [7], and, consequently, over the past two decades, a number of biological and physicochemical methods have been developed for remediation purposes [8,9]. In the particular case of biological processes, aerobic and anaerobic bacteria have attracted significant attention as the main active components for degradation of azo dyes [7]. Most recently, and in effort to improve the bioremediation efficiency and overall sustainability, numerous research efforts have focused on the development of alternative enzyme-based processes for treatment of textile effluents [10,11]. Despite being greener alternatives, these processes tend to be expensive due to the lack of reusability and the high sensitivity of enzymes under the conditions of conventional wastewaters (e.g., low pH, presence of denaturants, and high temperatures) [12,13]. Enzymes from different families have been used for bioremediation of dyes, including azoreductases, lignin peroxidases, and laccases [14].

Laccases (E.C. 1.10.3.2) are multicopper enzymes that catalyze the oxidation of phenolic and non-phenolic compounds by reducing an oxygen molecule to water [15]. In comparison with their bacterial counterparts, laccases from fungi sources show high versatility as a result of their affinity to a wide range of substrates and high redox potential at the T1 copper [16]. However, the lack of proper downstream unit operations for full laccase recovery after use for wastewater treatment can be expensive [17]. To overcome the recoverability, reusability, and stability issues, laccase immobilization has been considered as an attractive route [18,19]. Enzymatic immobilization can be performed through physical, covalent, or affinity interactions [20], among which covalent immobilization leads to superior thermal and storage stability, ease for downstream recovery, and very often increased regio- and stereo-selectivity [21,22]. Some of the preferred materials for immobilization include macroscopic materials, such as metal oxides, natural polymers, and some minerals, as well as nanomaterials, such as magnetite, gold, and silica [11,21,23,24]. Other recent reports have demonstrated that by immobilizing laccases, the rate of dye degradation increases considerably [25,26]. For instance, the immobilization of laccase on materials, such as Zirconia-Silica Hybrid Doped with Cu^+2^, is an attractive route for decolorization of the Remazol Brilliant Blue R dye [25]. This was also the case after immobilization of laccase on electrospun fibers of poly(methyl methacrylate)/polyaniline [26].

Despite the benefits of immobilization from the processing viewpoint, a major concern is to assure that, upon immobilization, no detrimental changes are induced in the secondary and tertiary structure of the enzyme molecules [27,28]. These detrimental changes are responsible for altering the active sites and consequently, for a significant reduction in the catalytic competency of the prepared immobilizates [29]. To maintain the structural stability, various strategies have been implemented including the use of surface fillers and spacers prior to immobilization (generally polymers), the immobilization at surface coverage regimes that promote protein-protein interactions over surface-protein interactions, and selection of suitable immobilization conditions (e.g., reaction time, pH, and temperature) [29]. To monitor changes in conformation, several methods have been implemented, including spectroscopy, as well as thermal and microscopy techniques [30]. For instance, through Fourier Transform Infrared (FTIR) spectroscopy, the conformational stability of immobilized enzymes has been estimated by deconvoluting the spectra of the immobilizates with the aid of the second derivative [31,32]. With this approach, it is possible to identify secondary structural changes induced by unfolding processes [31,32].

From the processing viewpoint, enzyme immobilizates have been incorporated into different types of reaction systems with the intention of maximizing their biotransformation capabilities. This superior performance, in turn, might lead to more cost-effective processes and the possibility for facilitated routes for scaling up if needed [33]. The reaction systems that have been explored for biotransformations with immobilized enzymes range from continuous stirred tank reactors to bubble columns [34]. The selection of the system depends on several parameters, including fluid properties, catalyst type, and diffusion limitations [34]. In the case of bioremediation processes, some of the reactor configurations include packed bed, fluidized bed, or even filters with trapped enzyme immobilizates (i.e., biofilters) [35,36,37]. One example is the use of fluidized bed reactors in the treatment of wastewater [38]. The use of biofilters has gained attention over the past few years due to the ease of assembly and operation, and the high remediation yield [39,40]. Additionally, these systems can retain other possible contaminants present in the effluents. The treatment processes can be conducted either in a batch or in a continuous mode [41].

One of the preferred choices for the treatment of effluents from the textile industry is the use of packed bed reactors with immobilized laccases with the main goal of achieving their decolorization. This continuous flow configuration might improve the contact time of the wastewater with the biocatalyst if sufficient surface area is provided by the packing material. Biofilters have been considered as an attractive alternative to increase the contact time for a robust continuous operation while maintaining the capital costs at a relatively low level [35]. To enable this technology, immobilized enzymes have been incorporated into fabrics or fibers by physical entrapment or covalent conjugation [26,42]. Further improvement of the remediation process has been achieved by conducting immobilization on nanostructured materials prior to their entrapment into the filter material [43]. In this regard, some of the immobilization supports that have been explored include gold nanoparticles, magnetic nanoparticles, silica nanoparticles, and graphene [42,44,45]. Despite the progress made to develop more reliable processes based on biofilters equipped with enzyme-based nanocomposites, a detailed understanding of the interplay of parameters for an optimized operation is still elusive.

Here, we present the treatment of textile-based filters with immobilized laccase from *Pycnoporus sanguineus* CS43 on silica nanoparticles for the degradation of Congo Red. We explored whether combining enzyme-based nanocomposites with silica-based nanoparticles known to alter the wetting phenomena of textiles [46] would increase overall decolorization. Laccase immobilization and synthesis of the nanocomposites were confirmed via Fourier-transform infrared (FTIR) spectroscopy. Changes in the secondary structure of the protein were inspected through the second derivative of the FTIR spectra. Six different filters were fabricated and tested in a continuous flow bioreactor in terms of their decolorization capabilities of Congo Red. Backscattered electrons (BSE) images of the different filters were obtained before and after the decolorization process. Percentage of decolorization and activity loss were determined as a function of time until a plateau in decolorization activity was reached. Experimental data was used as starting point to calibrate a multi-physics model of the filtering process. This allowed us to simulate different configurations of the filtering cartridge to determine the proper combination of parameters to maximize degradation of the dye. Our findings suggest that the treatment of textile-based filters with immobilized laccase in conjunction with hygroscopic nanocomposites provides a suitable avenue to achieve more efficient laccase dye decolorization than that obtained with similar filters treated only with free laccase. Previous reports have mainly focused on the potential of nanomaterials and textiles for immobilization separately and at a laboratory scale. Here, the synergy and superior performance of these two technologies at a bench scale facilitates the translation into pilot or even semi-industrial scales with higher chances of success.

## 2. Materials and Methods

### 2.1. Materials and Reagents

All materials were used as received and with no further purification. Acetone, chloro-trimethyl-silane (CTS), γ-amino(propyl) triethoxy silane (APTES), 2,2′-azino-bis(3-ethylbenzothiazoline-6-sulfonic acid) diammonium salt (ABTS), and glutaraldehyde were purchased from Sigma Aldrich, St. Louis, MO, USA. Hydrochloric acid (HCl), sodium hydroxide (NaOH), potassium dihydrogen phosphate (KH2PO4), and po tassium hydrogen phosphate (K_2_HPO_4_) were purchased from Merck, (Darmstadt, Germany). Ninety-one percent Congo Red was purchased from Matheson Coleman & Bell (Hatfield, PA, USA).

### 2.2. Laccase Production and Purification

Laccase enzyme was extracted from *P. Sanguineus* CS43 from tomato medium following the procedure in Reference [47]. In brief, two tangential filters with pore sizes of 0.5 mm and 0.2 mm in series were used to filtrate the mycelia from the culture supernatant. A membrane with a cut-off 10 kDa was used to ultrafiltrate the obtained laccase cocktail. Both laccases showed high amino acid sequence similarity (91%), and high thermostability at 50 °C and 60 °C, with half-lives of 277.7 h and 18 h for Lac I, and 35.8 h and 2.25 h for Lac II.

### 2.3. Enzyme Characterization

Two abundant isoforms, named LacI and LacII hereafter, were purified by ultrafiltration, ion exchange (IEX), and hydrophobic interaction chromatography, giving activities close to 285 U mg^−1^. SDS-electrophoresis was used to determine the molecular weights of LacI and LacII, which were found to be 68 kDa and 66 kDa, respectively. At acidic pH conditions, substrates, such as ABTS, 2,6-dimethoxy-phenol (DMP) and guaiacol, were oxidized by both isoforms. ABTS showed high specificity constants of 74,816 mM^−1^ s^−1^ and 36,746 mM^−1^ s^−1^ for LacI and LacII, respectively. At pH 3, Michaelis constants (Km) were found to be of 6.9 µm and 12.2 µm, respectively [48].

### 2.4. Enzymatic Activity Assay for Laccase

Laccase activity measurements were conducted in accordance to the method reported by Niku-Paavola et al. [49]. pH 4.0 phosphate buffer solution was prepared from KH_2_PO_4_ and K_2_HPO_4_. Kinetic spectrophotometric measurements were performed in a Genesis 10S spectrophotometer (Thermo Fisher Scientific, Walthman, MA, USA) for 2 min at 436 nm. One activity unit was defined as the amount of laccase needed to oxidize 1 µmol of ABTS per min. Laccase activity was expressed in terms of units per liter (U L^−1^) and was set at 1100 U L^−1^.

### 2.5. Synthesis of the Nanocomposites

Two types of nanocomposites were synthesized as described in Reference [46]. In brief, SiO_2_ nanoparticles were silanized in both milli-Q water and acetone by self-assembly method. Functionalized nanoparticles were dried by solvent evaporation method until a powder was obtained. Hereafter, silanized SiO_2_ nanoparticles prepared in milli-Q water are called NW, whereas those prepared in acetone are called NA. Effective silanization of both NW and NA was confirmed by FT-IR spectroscopy using an A250/D FTIR-ATR (Bruker, Billerica, MA, USA). These nanocomposites were selected due to their different hygroscopic properties, as reported in Reference [46].

### 2.6. Laccase Immobilization and Activity Measurements

**Figure 1:**
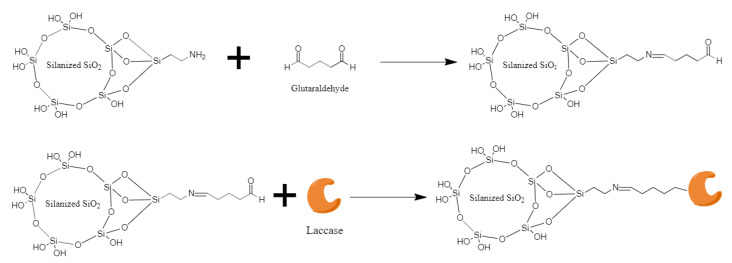
Reaction mechanism for enzyme immobilization. Upper panel shows the activation of NH_2_ terminal groups silanized silica nanoparticles with the aid of glutaraldehyde. Bottom panel shows the conjugation of laccase by forming imine bonds with the pre-activated silica nanoparticles.

Laccase immobilization on 100 nm SiO_2_ nanoparticles was conducted by covalent binding with the aid of glutaraldehyde as crosslinker [50,51]. In brief, 200 µL of 20% (*v*/*v*) APTES solution was added to a 0.583% (*w*/*v*) SiO_2_ nanoparticles solution in acetone and stirred overnight to promote silanization. The solution was placed in a desiccator until a powder was obtained. Silanization was confirmed by Fourier transform infrared spectroscopy (FT-IR) on a A250/D FTIR-ATR (Bruker, United States). One gram of silanized SiO_2_ nanoparticles were dissolved in 10 mL of 2% (*v*/*v*) glutaraldehyde solution and let to react for 2 h at room temperature. The excess of glutaraldehyde was removed by filtration using a filter with pore size of 0.2 µm and several washes with milli Q water. The obtained powder was mixed with 2 mL of milli Q water and 1 mL of laccase solution (40,000 U L^−1^). A schematic of the reaction mechanism of immobilization is shown in Figure 1. Effective immobilization was confirmed by laccase activity measurements following the same procedure described for free laccase. Immobilized activity was adjusted to 1100 U L^−1^.

### 2.7. Bioreactor Fabrication and Decolorization Measurements

**Figure 2:**
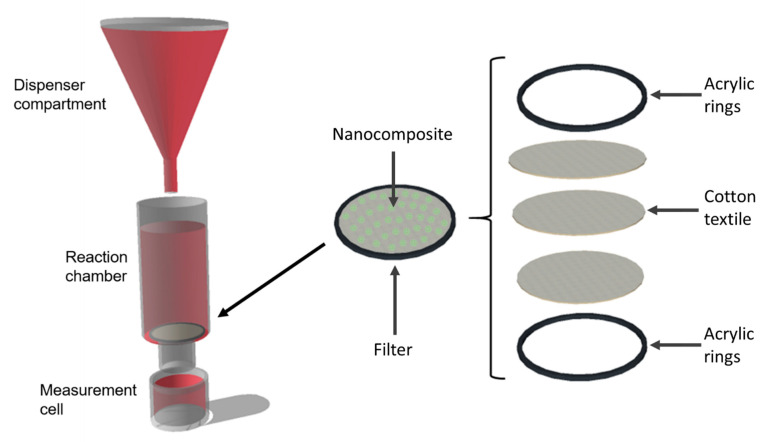
Bioreactors schematic. The bioreactors consisted of a dispenser compartment, a reaction chamber and a measurement cell. The reaction chamber contained a textile-based biofilter with free or immobilized laccase molecules as biocatalysts. The dispenser compartment was set to release a drop of the artificial wastewater every second.

Six different bioreactors were designed and assembled to study decolorization of model wastewaters with the aid of biofilters with both free and immobilized laccase in combination with silanized silica nanoparticles. Bioreactors consisted of a dispenser compartment, a reaction chamber, and a measurement cell as shown in Figure 2.

The dispenser compartment contained artificial wastewater solution prepared from the dilution of Congo Red in milli-Q water (0.7% (*w*/*v*)), which was kept in the dark and at room temperature until analysis. Dye characteristics are shown in Table 1. Congo Red was released into the reaction chamber drop by drop at an average speed of 1 drop per second.

The reaction chamber contained a textile-based filter for decolorization of the artificial wastewater. Filters were fabricated from the stock of three layers of 100% cotton textile with a diameter of 3.2 cm and were maintained tighten between two acrylic rings. Five hundred microliters of the desired solution were placed on the filter and left to dry overnight prior to each experiment. Six different filters were tested by duplicate to attain their decolorization properties. Biofilters were fabricated from the deposition of free laccase (Free), immobilized laccase on SiO_2_ nanoparticles (IL), free laccase and NW (FreeNW), free laccase and NA (FreeNA), immobilized laccase on NW SiO_2_ nanoparticles (ILNW), and immobilized laccase on NA SiO_2_ nanoparticles (ILNA), on the surface of the textile [46,52]. The measurement cell collected the residual dye after it passed through the filter. The cell had enough volume for spectrophotometric measurements every five minutes and was used to determine the percentage of decolorization and the cumulative amount of laccase units lost during the filtering process. Residual dye concentration was calculated by measuring the area under the curve of the absorbance spectrum between 350 and 700 nm, using the following equation [53]:

(1) $$\%\;\mathrm{decoloration}=\frac{A_{i}-A}{A_{i}}\times 100,$$

where A_i_ is the area under the curve for the untreated dye, and A the measured area. The cumulative amount of laccase units lost during the filtering process was calculated by conducting laccase activity measurements as described in Section 2.4.

### 2.8. Scanning Electron Microscopy (SEM) of the Textile-Based Filters

Backscattered electrons (BSE) images of the decolorization biofilters were obtained before and after decolorization experiments under a JSM-6490LV scanning electron microscope (SEM) (JEOL, Tokio, Japan). Prior to observation, the filters were gold-coated under a Desk IV sputter coater (Denton Vacuum, Moorestown, NJ, USA) to obtain a layer of 50 nm.

### 2.9. Simulation and Mathematical Model of Decolorization Filters

Experimental data was used to calibrate a multi-physics model of the biofilters. All simulations and biofilter configurations were implemented in COMSOL Multiphysics^®^ 5.3 (Stockholm, Sweden). Transport of Congo Red through the filter system was modeled with the aid of the Transport of Diluted Species in Porous Media Module. The equations describing the transport of Congo Red are shown below (Equations (2)–(7)), which correspond to the Brinkman equations for species transport in porous media. Biofilters were set with a density of 1.5 × 10^3^ kg m^−3^ and a porosity factor of 0.5. Simulations were performed in a 2D model of the system to simplify calculations and reduce computational time. Diffusion and dispersion coefficients were unique for each of the filters, namely Free, IL, FreeNW, FreeNA, ILNW, and ILNA. Biofilters were simulated as made of three rectangular computational subdomains of 32 mm × 0.5 mm each:

(2) $$P_{1j}\frac{{\delta c}_{i}}{\delta t}+P_{2j}+\nabla \cdot \Gamma_{i}=R_{i}+S_{i},$$

(3) $$P_{1j}=\left(\epsilon_{p}+{\rho k}_{\mathrm{pj}}\right),$$

(4) $$P_{2j}=\left(c_{i}-\rho_{p}c_{\mathrm{pj}}\right)\frac{\delta \epsilon_{p}}{\delta t},$$

(5) $$N_{i}=\Gamma_{i}=-\left(D_{\mathrm{Dj}}+D_{\mathrm{ej}}\right)\nabla c_{i},$$

(6) $$D_{\mathrm{ej}}=\frac{\epsilon_{p}}{\tau_{\mathrm{Fj}}}D_{\mathrm{Fj}},$$

(7) $$\tau_{\mathrm{Fj}}=\epsilon_{p}^{-\frac{1}{2}},$$

where *D_f_* corresponds to the diffusion constant of the filter, *D_d_* to the dispersion constant of the filter, R_i_ to the rate of reaction between the dye and the enzyme, ϵ_p_ to the porosity of the material, ρ to the density of the material, c_i_ to the dye concentration, D_e_ to the diffusion through the material, and S_i_ to the inflow of the dye.

**Figure 3:**
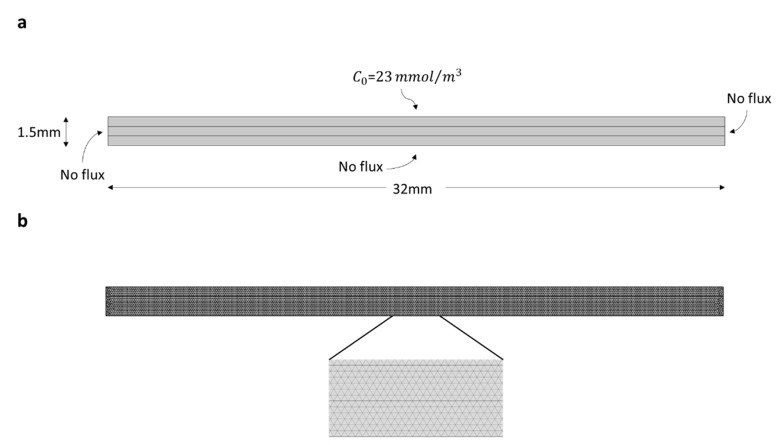
Computational domain in COMSOL Multiphysics^®^. (**a**) Dimensions and boundary conditions and (**b**) mesh distribution with 3000 triangular elements.

A constant concentration of Congo Red dye (23 mmol m^−3^) on the surface of the filter was assumed to simulate the continuous dropping of this dye applied experimentally. A flow velocity of 10 mm s^−1^ was used for the simulations. Reaction rates used for the simulations were obtained directly from experimental data. To be able to compare simulated results with experimental data, simulations were carried out for 90 min of reaction with a sampling period of 5 min. An initial condition of no Congo Red in the computational domain was assumed. The computational domain and boundary conditions are shown in Figure 3a. A mesh convergence analysis showed that 3000 triangular elements were needed to obtain robust results (Figure 3b).

The appropriate operating parameters to match simulations and experimental results on decolorization were found by trial and error. The parameters that were changed were Diffusion (*D_f_*) and dispersion (*D_d_*). Once the process was properly described by the simulation results, new configurations combining different filter arrangements were setup and simulated to find the combination that produces the greatest removal of the dye.

## 3. Results

### 3.1. Immobilization and Characterization of the Nanocomposites

**Figure 4:**
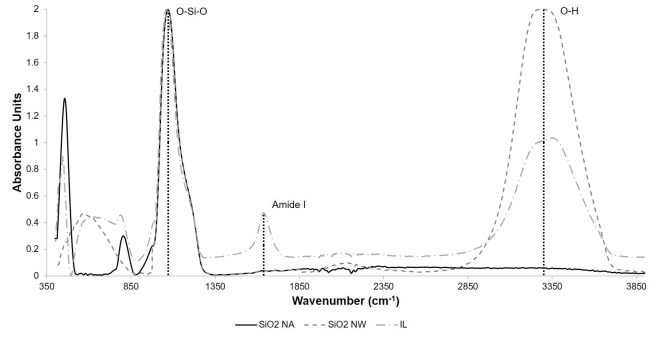
FT-IR analysis of silanized nanoparticles and immobilizates silica-laccase. The presence of the peak at 1087 cm^−1^ corresponds to the O-Si-O bond and corroborates effective silanization. Immobilization of laccase (nanocomposite IL) was confirmed by the presence of the amide I band at around 1640 cm^−1^.

Silanization of silica nanoparticles was confirmed by FT-IR analysis. Figure 4 shows the average spectrum of five independent FT-IR analysis of NW and NA. In both cases, it is possible to observe the O-Si-O bonding characteristic of nanoparticles silanization around 1087 cm^−1^. Nanoparticles prepared in acetone media were used to immobilize free laccase. Correct attachment of the enzyme was confirmed by the appearance of the Amide I bond through at least five independent FT-IR analyses (Figure 4).

**Figure 5:**
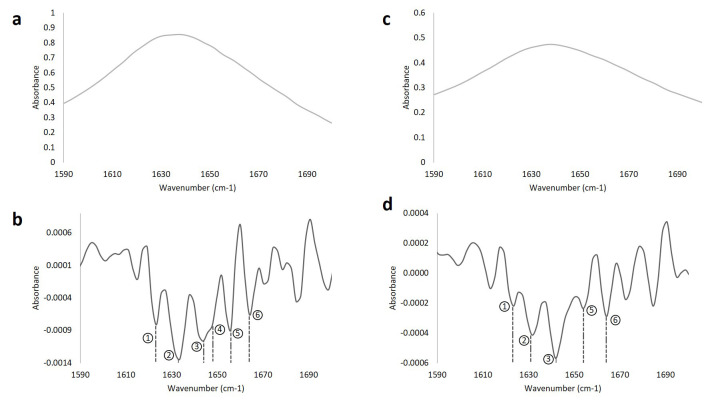
Average FT-IR and second derivative analysis of the amide I bond. (**a**) Amide I bond of free laccase, (**b**) second derivative of the amide I bond of free laccase, (**c**) amide I bond of immobilized laccase (IL), and (**d**) second derivative of the amide I bond of IL. The numbers 1, 2, and 3 correspond to change in the β-sheet structure. Number 4 corresponds to Random coil. Number 5 and 6 correspond to α-helix and 310-helix structure, respectively.

The second derivative of the Amide I bond was calculated using the Savitsky-Golay method to evaluate protein secondary structure prior and after immobilization as stated in Reference [54]. Figure 5 shows the obtained derivatives prior and after immobilization. Assignment of the secondary structural components for laccase was performed based on the values reported in Reference [55] and are summarized in Table 2.

### 3.2. Decolorization and Activity Measurements

**Figure 6:**
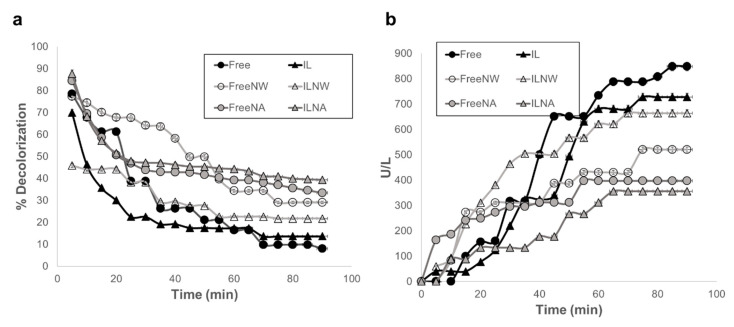
Decolorization and residual activity measurements of Free, IL, FreeNW, ILNW, FreeNA, and ILNA. (**a**) Percentage of decolorization as a function of time and (**b**) cumulative enzymatic activity (U L^−1^) of the artificial wastewater after the decolorization treatment. NW = silanized SiO_2_ nanoparticles prepared in milli-Q water; NA = silanized SiO_2_ nanoparticles prepared in acetone.

The ability of free and immobilized laccase to decolor artificial wastewaters in the presence of the different nanocomposites was tested with Congo Red solutions. Measurements were recorded in continuous flow to determine the loss of transformation ability at different times until the filters reached a plateau. Figure 6 shows the percentage of decolorization of Free, IL, FreeNW, ILNW, FreeNA, and ILNA as a function of time (a) and the cumulative enzymatic activity loss after every cycle (b). Although all decolorization filters started from the same amount of enzyme activity, our findings suggest that the interaction of free and immobilized laccase with NA had a greater percentage dye removal after plateau. After 1 h, percentages of decolorization leveled off at 39.4% for ILNA, 33.4% for FreeNA, 29.1% for FreeNW, 21.7% for ILNW, 13.7% for IL, and 8.1% for Free. All the data is summarized in Supplementary Table S1.

**Figure 7:**
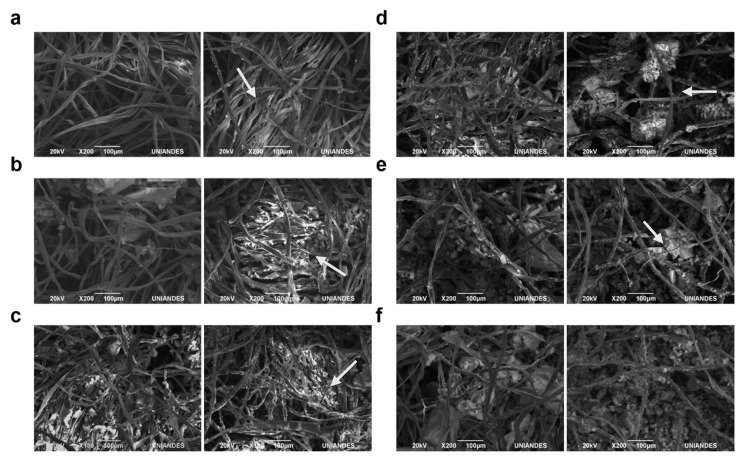
Backscattered electron (BSE) images of the biofilters before and after decolorization assays. (**a**) before (left) and after (right) of Free, (**b**) before (left) and after (right) of IL, (**c**) before (left) and after (right) of FreeNW, (**d**) before (left) and after (right) of ILNW, (**e**) before (left) and after (right) of FreeNA, and (**f**) before (left) and after (right) of ILNA. White arrows point to deposits after decolorization. These are most likely attributed to byproducts of the reaction that deposit along the fibers of the filter.

The morphology and composition of the biofilters was evaluated via BSE imaging. High molecular weight byproducts are likely observed along cotton fibers after decolorization, as evidenced by the presence of lighter areas in Figure 7.

### 3.3. Multiphysics Modeling and Simulation of the Decolorization Process

**Figure 8:**
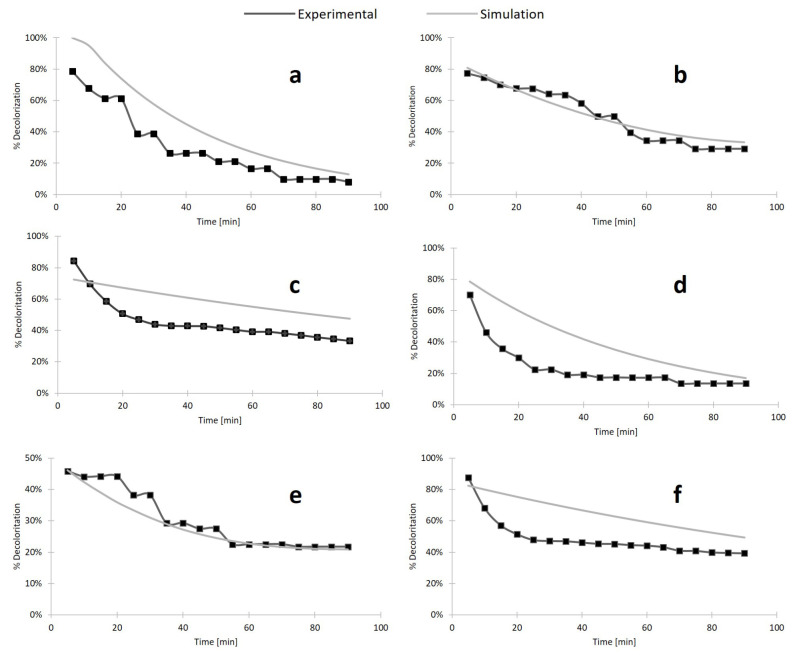
Experimental and simulation results of (**a**) Free filters, (**b**) FreeNW filters, (**c**) FreeNA filters, (**d**) IL biofilters, (**e**) ILNW biofilters, and (**f**) ILNA biofilters. Simulation results were used to determine the diffusion and dispersion constants of the Brinkman equations.

**Figure 9:**
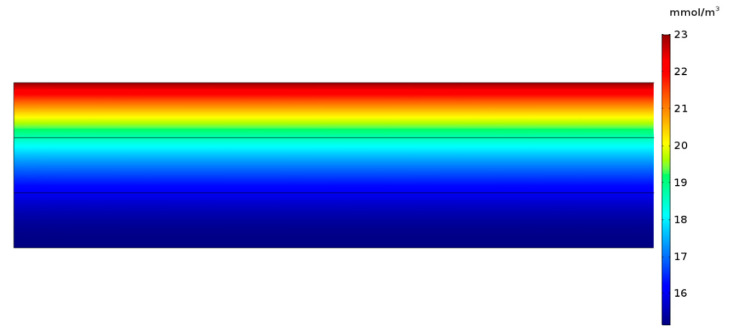
Dye degradation concentration profile in COMSOL Multiphysics^®^. As the dye diffuses through the biofilter, it is consumed until it reaches a constant concentration.

Simulations were carried out varying the diffusion and dispersion parameters for each filter until results were comparable with experimental data. Obtained decolorization curves for each of the designed filters can be found in Figure 8. Figure 8a,b,e show that simulation results agree well with the obtained experimental data. This was not the case for Figure 8c,d,f, where we found significant differences between the two datasets. We hypothesize that this could be due to the increased hydrophobicity of such biofilters, which alters the contact time between the laccase molecules and the dye. This leads to a two-stage degradation process where the dye is initially biocatalyzed rapidly followed by a regime that proceeds with an approximately constant degradation rate. This two-stage process requires a more sophisticated modeling approach that goes beyond the scope of this contribution. An example of the dye concentration profile during a typical degradation experiment is shown in Figure 9.

Simulations allowed the description in terms of *D_f_* and *D_d_* of the transport of Congo Red of the different filters. The variability in diffusion and dispersion across the filters suggests that these parameters have a slight dependence on the immobilization method. Nonetheless, their variability was within one order of magnitude in all cases. Table 3 summarizes the recovered values of these parameters for each type of filter.

The calibrated mathematical model of each type of filter was further used to study the effects of combining the biofilters in series within the filter cartridge. Decolorization results for the different combinations are shown in Supplementary Figures S1–S16. According to our simulations, the combination of biofilters ILNA and FreeNW shows the most promising results, reaching a plateau of decolorization of 78% and maintaining a removal of 100% for about 50 min. In addition, all combinations seemed to provide a better removal than Free and IL filters and leaving the Free-IL combination as the least efficient, thereby suggesting that interaction with the nanocomposites enhances the removal capabilities of the different biofilters.

## 4. Discussion

In this study, we report the usage of laccase immobilization and subsequent incorporation into cotton filters for the continuous treatment of wastewaters from the textile industry. Our results suggest that the combination of filters with different wettability and immobilization strategies can be used to maximize Congo Red degradation while minimizing enzymatic loss. For laccase immobilization, silanization was sufficient as demonstrated by the presence of the Si-O-Si and Amide I bond in the FTIR spectrum of the modified nanoparticles (Figure 4). The two nanocomposites, namely NW and NA, were then tested as active components of the decolorization filters assembled in a continuous system. Although both nanocomposites differed only in their synthesis solvent, namely water and acetone, their wetting phenomena when included in cotton fabric was drastically altered, as reported previously in Reference [46]. NW nanocomposites produced a hydrophilic behavior, while the NA ones conferred a temporal hydrophobicity to the textiles. This unique property resulted in longer retention times for each drop of water in the textile, thereby leading to longer interaction between the dye and the immobilized laccase molecules. This might be the reason why both FreeNA and ILNA filters showed the highest percentage of decolorization (33% and 39%, respectively) after reaching a plateau. Treatments leading to the lowest levels of dye removal were IL and Free (14% and 8%, respectively). These results confirm that by combining immobilized laccase with the nanocomposites, the percentage of dye decolorization increased with respect to free laccase and just immobilized laccase in the filter. This synergistic behavior has been previously described for systems where immobilized lipase enzyme from *Candida rugosa* was applied for the lipolytic activity in conjunction with gold nanoparticles [57,58]. In this study, the improved behavior was attributed to the hydrophobic/hydrophilic interaction of the nanoparticles [57]. Similar studies demonstrated dye removal efficiencies in the range of 30% to 100% [25,26,59]. However, such levels of removal have been mainly achieved on batch processes with longer removal times than ours (i.e., in the scale of hours). Our approach allows to increase the residence time of the dye within the biofilters, which led to reach removal efficiencies close to those reported in the literature in a time scale of a few minutes.

The second derivative of the Amide I bond from the FTIR spectra of at least five independents assays was calculated to identify changes in the secondary structure of laccase after immobilization. This is an effective method towards measuring changes in the secondary structure due to the high sensitivity of the Amide I bond to variations in molecular geometry and hydrogen bonding [55]. According to our results, upon immobilization, the secondary structure of laccase molecules suffered a subtle reduction in the contents of α-helical components, while β-sheets remained approximately constant. This low level of secondary conformational changes allows to maintain a sufficient number of catalytically active laccase molecules. Moreover, the results confirm that the laccase molecules are flexible enough to prevent undergoing detrimental structural changes upon interactions with surfaces. This seems to be related to the presence of a high amount of random coils in the laccase structure, which is around 40% [56].

After operation within the bioreaction system, the biofilters were removed and imaged with the aid of BSE. The presence of large deposits along the fibers of the filters were attributed to the presence of reaction byproducts. Such deposition during biofilter operation have been reported previously for similar systems [60,61]. FreeNA and ILNA exhibited a considerably lower concentration of the byproducts along the cotton fibers after decolorization (Figure 7e,f). This is consistent with the removal results, where higher levels of dye degradation were evident. The production of these compounds could be detrimental for the overall performance of the system in the long run and should be considered with care for further scaling-up processes.

The possibility of maximizing the removal efficiencies by changing the arrangement of the filters was explored by calibrating a multi-physics simulation with the experimental results. The diffusion and dispersion coefficients were used to adjust the simulation as closely as possible to the observed degradation results. These parameters were chosen as they have been shown to alter the transport of species in porous media [62]. According to our results, the best decolorization results are achieved for combinations of filters with immobilized enzymes and nanocomposites prepared in the presence of acetone. This was attributed to the increased hydrophobicity of such materials, which increases the residence time of the aqueous solution containing the dye within the filtration system. Interestingly, this seems to be the case despite the diffusion and dispersion coefficients staying within one order of magnitude between the filters, suggesting that degradation in the filters is governed mainly by microscopic properties. Similar results have been reported for biofilters superficially treated with different hydrophobic molecules, such as fluorodiazomethanes, trimethylchlorosilane, and hexamethyldisilazane [63,64,65]. This property is advantageous for long-term operation as it potentially helps in avoiding the early clogging of the biofilters by the presence of suspended particulate material [66].

## 5. Conclusions

The treatment of effluents from the textile industry has been challenging due to the presence of compounds, such as dyes, which tend to be recalcitrant and usually require advanced multistage treatment processes. This approach has led to intermediate removal efficiencies at the expense of considerable capital investments in sophisticated equipment to operate under extreme conditions (e.g., high temperatures and pressures). Due to the increasingly stringent environmental regulatory framework worldwide, a number of strategies have pushed research for more efficient and cost-effective remediation processes. One of such initiatives is the use of highly-active enzymes, such as laccases, as agents for the degradation of dyes. These alternative-green processes operate under mild conditions but still face challenges associated with the stability of enzymes in wastewaters. Here, we attempted to address this issue by immobilizing laccase molecules on silanized-silica nanoparticles and its subsequent entrapment in textile filters to extend the activity of the enzyme molecules while incorporated in a continuous remediation process. Laccase-free, silanized-silica nanoparticles were used as negative controls. The prepared biofilters were tested with a model wastewater containing Congo red dye and showed up to 40% removal when the nanoparticles were synthesized in acetone. This was attributed to an increased hydrophobicity that is likely to extend the residence time and the interaction with the laccase molecules of the dye. The experimental results were used to calibrate a Multiphysics transport model in COMSOL Multiphysics^®^ to evaluate the removal efficiency of different filter configurations. According to our simulations, filters with immobilized enzymes and nanocomposites prepared in the presence of acetone seem to be the most efficient for removal. Our encouraging results strongly suggest that biofilters with deposited laccase-silica nanocomposites might be a suitable avenue for highly-effective, continuous bioremediation processes. Additional testing in conditions closer to those found in wastewaters, and larger-scale studies will be required for a more conclusive assessment of our biofilters.

## Figures and Tables

**Table 1:** Main features of Congo Red, the dye used here to study decolorization. The red circles show the azo dye chromophores.

Dye	λmax (nm)	Color Index Number	Color Index Name	Structure
Congo Red	500	22120	Direct Red 28	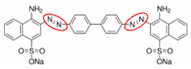

**Table 2:** Band assignments for free and immobilized laccase in the Amide I infrared region based on Reference [56].

Wavenumber [cm^−1^]		Assignment
Free Laccase	Immobilized Laccase	
1623	1623	β-sheet
1633	1631	β-sheet
1644	1642	β-sheet
1648	-	Random coil
1656	1654	α-helix
1664	1664	3_10_-helix

**Table 3:** Diffusion and dispersion parameters as recovered from the simulations of the different types of filters. Values were found by trial and error to match the simulation results with the experimental data for decolorization.

Type of Filter	Parameter	
	*D_f_*	*D_d_*
Free	2.5 × 10^−6^	1 × 10^−9^
FreeNW	2.6 × 10^−6^	2 × 10^−9^
FreeNA	2.6 × 10^−6^	2 × 10^−9^
IL	3.2 × 10^−6^	2 × 10^−9^
ILNW	4.7 × 10^−6^	2 × 10^−9^
ILNA	2.7 × 10^−6^	2 × 10^−9^

## Supplementary Materials

The following are available online at https://www.mdpi.com/2079-4991/10/6/1227/s1, Table S1: Decolorization percentage and residual activity measurements of Free, IL, FreeNW, ILNW, FreeNA and ILNA, Figure S1: Simulation results of combining IL and ILNW filters, Figure S2: Simulation results of combining FreeNW and ILNW filters, Figure S3: Simulation results of combining FreeNW and IL filters, Figure S4: Simulation results of combining ILNW and ILNA filters, Figure S5: Simulation results of combining IL and ILNA filters, Figure S6: Simulation results of combining FreeNW and ILNA filters, Figure S7: Simulation results of combining FreeNA and ILNW filters, Figure S8: Simulation results of combining FreeNA and IL filters, Figure S9: Simulation results of combining FreeNA and FreeNW filters, Figure S10: Simulation results of combining FreeNA and ILNA filters, Figure S11: Simulation results of combining Free and ILNW filters, Figure S12: Simulation results of combining Free and IL filters, Figure S13: Simulation results of combining Free and FreeNW filters, Figure S14: Simulation results of combining Free and ILNA filters, Figure S15: Simulation results of combining Free and FreeNA filters, Figure S16: Simulation results of all two-combinations of the different types of filters.

## References

[1] Rovira J., Domingo J.L. (2019). Human health risks due to exposure to inorganic and organic chemicals from textiles: A review. Environ. Res..

[2] Tang A.Y.L., Lo C.K.Y., Kan C. (2018). Textile dyes and human health: A systematic and citation network analysis review. Color. Technol..

[3] Chung K.-T. (2016). Azo dyes and human health: A review. J. Environ. Sci. Health Part C.

[4] Nahar K., Chowdhury M.A.K., Chowdhury M.A.H., Rahman A., Mohiuddin K.M. (2018). Heavy metals in handloom-dyeing effluents and their biosorption by agricultural byproducts. Environ. Sci. Pollut. Res..

[5] Sivaram N.M., Gopal M. (2019). Toxic Waste From Textile Industries. Energy from Toxic Organic Waste for Heat and Power Generation.

[6] Martins L.O., Soares C.M., Pereira M.M., Teixeira M., Costa T., Jones G.H., Henriques A.O. (2002). Molecular and Biochemical Characterization of a Highly Stable Bacterial Laccase That Occurs as a Structural Component of the *Bacillus subtilis* Endospore Coat. J. Biol. Chem..

[7] Stefanakis A., Akratos C.S., Tsihrintzis V.A. (2014). Vertical Flow Constructed Wetlands: Eco-Engineering Systems for Wastewater and Sludge Treatment.

[8] Aravind P., Selvaraj H., Ferro S., Sundaram M. (2016). An integrated (electro- and bio-oxidation) approach for remediation of industrial wastewater containing azo-dyes: Understanding the degradation mechanism and toxicity assessment. J. Hazard. Mater..

[9] Khattab T.A., Abdelrahman M.S., Rehan M. (2019). Textile dyeing industry: Environmental impacts and remediation. Environ. Sci. Pollut. Res..

[10] Enayatzamir K., Alikhani H.A., Couto S.R. (2009). Simultaneous production of laccase and decolouration of the diazo dye Reactive Black 5 in a fixed-bed bioreactor. J. Hazard. Mater..

[11] Rodríguez-Couto S. (2012). A promising inert support for laccase production and decolouration of textile wastewater by the white-rot fungus Trametes pubescesns. J. Hazard. Mater..

[12] Zheng G., Liu S., Zha J., Zhang P., Xu X., Chen Y., Jiang S. (2019). Protecting Enzymatic Activity via Zwitterionic Nanocapsulation for the Removal of Phenol Compound from Wastewater. Langmuir.

[13] Chiong T., Lau S.Y., Lek Z.H., Koh B.Y., Danquah M.K. (2016). Enzymatic treatment of methyl orange dye in synthetic wastewater by plant-based peroxidase enzymes. J. Environ. Chem. Eng..

[14] Bhatia D., Sharma N.R., Singh J., Kanwar R.S. (2017). Biological methods for textile dye removal from wastewater: A review. Crit. Rev. Environ. Sci. Technol..

[15] Barbosa N.L., Ornelas-Soto N., Osma J.F., Harris A. (2017). Uses of Laccase in the Monitoring and Treatment of Water and Food. Laccase Applications Investigations and Insights (USA).

[16] Pezzella C., Giacobbe S., Giacobelli V.G., Guarino L., Kylic S., Sener M., Sannia G., Piscitelli A. (2016). Green routes towards industrial textile dyeing: A laccase based approach. J. Mol. Catal. B Enzym..

[17] Arabaci G., Usluoglu A. (2014). The Enzymatic Decolorization of Textile Dyes by the Immobilized Polyphenol Oxidase from Quince Leaves. Sci. World J..

[18] Fernández-Fernández M., Sanromán M.Á., Moldes D. (2013). Recent developments and applications of immobilized laccase. Biotechnol. Adv..

[19] Conte M.P., Sahoo J.K., Abul-Haija Y.M., Lau K.H.A., Ulijn R.V. (2018). Biocatalytic Self-Assembly on Magnetic Nanoparticles. ACS Appl. Mater. Interfaces.

[20] Campaña A.L., Sotelo D.C., Oliva H.A., Aranguren A., Ornelas-Soto N., Cruz J.C., Osma J.F. (2020). Fabrication and Characterization of a Low-Cost Microfluidic System for the Manufacture of Alginate–Lacasse Microcapsules. Polymers (Basel).

[21] Datta S., Christena L.R., Rajaram Y.R.S. (2013). Enzyme immobilization: An overview on techniques and support materials. 3 Biotech.

[22] Patel S.K.S., Kalia V.C., Choi J.-H., Haw J.-R., Kim I.-W., Lee J.K. (2014). Immobilization of laccase on SiO₂ nanocarriers improves its stability and reusability. J. Microbiol. Biotechnol..

[23] Chakraborty S., Rusla H., Nath A., Sikder J., Bhattacharjee C., Curcio S., Drioli E. (2016). Immobilized biocatalytic process development and potential application in membrane separation: A review. Crit. Rev. Biotechnol..

[24] Stine K.J. (2017). Enzyme Immobilization on Nanoporous Gold: A Review. Biochem. Insights.

[25] Jankowska K., Ciesielczyk F., Bachosz K., Zdarta J., Kaczorek E., Jesionowski T. (2019). Laccase Immobilized onto Zirconia–Silica Hybrid Doped with Cu2+ as an Effective Biocatalytic System for Decolorization of Dyes. Materials (Basel).

[26] Jankowska K., Zdarta J., Grzywaczyk A., Kijeńska-Gawrońska E., Biadasz A., Jesionowski T. (2020). Electrospun poly(methyl methacrylate)/polyaniline fibres as a support for laccase immobilisation and use in dye decolourisation. Environ. Res..

[27] Singh P., Morris H., Tivanski A.V., Kohen A. (2015). Determination of concentration and activity of immobilized enzymes. Anal. Biochem..

[28] Bolibok P., Wiśniewski M., Roszek K., Terzyk A.P. (2017). Controlling enzymatic activity by immobilization on graphene oxide. Naturwissenschaften.

[29] Mateo C., Palomo J.M., Fernandez-Lorente G., Guisan J.M., Fernandez-Lafuente R. (2007). Improvement of enzyme activity, stability and selectivity via immobilization techniques. Enzyme Microb. Technol..

[30] Mohamad N.R., Marzuki N.H.C., Buang N.A., Huyop F., Wahab R.A. (2015). An overview of technologies for immobilization of enzymes and surface analysis techniques for immobilized enzymes. Biotechnol. Biotechnol. Equip..

[31] Schartner J., Guldehaupt J., Mei B., Rogner M., Muhler M., Gerwert K., Kotting C. (2013). Universal Method for Protein Immobilization on Chemically Functionalized Germanium Investigated by ATR-FTIR Difference Spectroscopy. J. Am. Chem. Soc..

[32] Kowalczuk D., Pitucha M. (2019). Application of FTIR Method for the Assessment of Immobilization of Active Substances in the Matrix of Biomedical Materials. Materials (Basel).

[33] (2017). Immobilized Biocatalysts. Biocatalysis.

[34] Liese A., Hilterhaus L. (2013). Evaluation of immobilized enzymes for industrial applications. Chem. Soc. Rev..

[35] El-Naas M.H., Acio J.A., el Telib A.E. (2014). Aerobic biodegradation of BTEX: Progresses and Prospects. J. Environ. Chem. Eng..

[36] Shalini P.S.Y. (2019). Multistage fluidized bed bioreactor for dye decolorization using immobilized polyurethane foam: A novel approach. Biochem. Eng. J..

[37] Chhabra M., Mishra S., Sreekrishnan T.R. (2015). Combination of chemical and enzymatic treatment for efficient decolorization/degradation of textile effluent: High operational stability of the continuous process. Biochem. Eng. J..

[38] Bello M.M., Raman A.A.A., Purushothaman M. (2017). Applications of fluidized bed reactors in wastewater treatment—A review of the major design and operational parameters. J. Clean. Prod..

[39] Chaudhary D.S., Vigneswaran S., Ngo H.-H., Shim W.G., Moon H. (2003). Biofilter in water and wastewater treatment. Korean J. Chem. Eng..

[40] Reungoat J., Escher B.I., Macova M., Keller J. (2011). Biofiltration of wastewater treatment plant effluent: Effective removal of pharmaceuticals and personal care products and reduction of toxicity. Water Res..

[41] Simon J., Wiese J., Steinmetz H. (2006). A Comparison of Continuous Flow and Sequencing Batch Reactor Plants Concerning Integrated Operation of Sewer Systems and Wastewater Treatment Plants. Water Sci. Technol..

[42] Goldhahn C., Burgert I., Chanana M. (2019). Nanoparticle-Mediated Enzyme Immobilization on Cellulose Fibers: Reusable Biocatalytic Systems for Cascade Reactions. Adv. Mater. Interfaces.

[43] Du Q., Sun J., Li Y., Yang X., Wang X., Wang Z., Xia L. (2014). Highly enhanced adsorption of congo red onto graphene oxide/chitosan fibers by wet-chemical etching off silica nanoparticles. Chem. Eng. J..

[44] Murugappan G., Zakir M.J.A., Jayakumar G.C., Khambhaty Y., Sreeram K.J., Rao J.R. (2016). A Novel Approach to Enzymatic Unhairing and Fiber Opening of Skin Using Enzymes Immobilized on Magnetite Nanoparticles. ACS Sustain. Chem. Eng..

[45] Zdarta J., Meyer A., Jesionowski T., Pinelo M. (2018). A General Overview of Support Materials for Enzyme Immobilization: Characteristics, Properties, Practical Utility. Catalysts.

[46] Barbosa N.-L., Osma J.F.F. (2017). Nanocomposites fabrication by self-assembly method to modify macroscopic properties. J. Phys. Conf. Ser.

[47] Ramírez-Cavazos L.I., Junghanns C., Ornelas-Soto N., Cárdenas-Chávez D.L., Hernández-Luna C., Demarche P., Enaud E., García-Morales R., Agathos S.N., Parra R. (2014). Purification and characterization of two thermostable laccases from Pycnoporus sanguineus and potential role in degradation of endocrine disrupting chemicals. J. Mol. Catal. B Enzym..

[48] Garcia-Morales R., Rodríguez-Delgado M., Gomez-Mariscal K., Orona-Navar C., Hernandez-Luna C., Torres E., Parra R., Cárdenas-Chávez D., Mahlknecht J., Ornelas-Soto N. (2015). Biotransformation of Endocrine-Disrupting Compounds in Groundwater: Bisphenol A, Nonylphenol, Ethynylestradiol and Triclosan by a Laccase Cocktail from Pycnoporus sanguineus CS43. Water. Air. Soil Pollut..

[49] Paavola M.L.N., Raaska L., Itavaara M. (1990). Detection of white-rot fungi by a non-toxic stain. Mycol. Res. (UK).

[50] Fang J., Böhringer K.F., Achour H. (2016). Self-Assembly. Ref. Modul. Mater. Sci. Mater. Eng..

[51] Mahalingam V., Onclin S., Péter M., Ravoo B.J., Huskens J., Reinhoudt D.N. (2004). Directed Self-Assembly of Functionalized Silica Nanoparticles on Molecular Printboards through Multivalent Supramolecular Interactions. Langmuir.

[52] Rodríguez Couto S., Osma J.F., Saravia V., Gübitz G.M., Toca Herrera J.L. (2007). Coating of immobilised laccase for stability enhancement: A novel approach. Appl. Catal. A Gen..

[53] Zare K., Sadegh H., Shahryari-ghoshekandi R., Maazinejad B., Ali V., Tyagi I., Agarwal S., Gupta V.K. (2015). Enhanced removal of toxic Congo red dye using multi walled carbon nanotubes: Kinetic, equilibrium studies and its comparison with other adsorbents. J. Mol. Liq..

[54] Cruz J.C., Pfromm P.H., Tomich J.M., Rezac M.E. (2010). Conformational changes and catalytic competency of hydrolases adsorbing on fumed silica nanoparticles: I. Tertiary structure. Colloids Surf. B Biointerfaces.

[55] Yang H., Yang S., Kong J., Dong A., Yu S. (2015). Obtaining information about protein secondary structures in aqueous solution using Fourier transform IR spectroscopy. Nat. Protoc..

[56] Kameshwar A.K.S., Barber R., Qin W. (2018). Comparative modeling and molecular docking analysis of white, brown and soft rot fungal laccases using lignin model compounds for understanding the structural and functional properties of laccases. J. Mol. Graph. Model..

[57] Fratoddi I. (2017). Hydrophobic and Hydrophilic Au and Ag Nanoparticles. Breakthroughs and Perspectives. Nanomaterials.

[58] Cao-Milán R., He L.D., Shorkey S., Tonga G.Y., Wang L.-S., Zhang X., Uddin I., Das R., Sulak M., Rotello V.M. (2017). Modulating the catalytic activity of enzyme-like nanoparticles through their surface functionalization. Mol. Syst. Des. Eng..

[59] Li Z., Chen Z., Zhu Q., Song J., Li S., Liu X. (2020). Improved performance of immobilized laccase on Fe3O4@C-Cu2+ nanoparticles and its application for biodegradation of dyes. J. Hazard. Mater..

[60] Warsinger D.M., Chakraborty S., Tow E.W., PLumlee M.H., Bellona C., Loutatidou S., Karimi L., Mikelonis A.M., Achilli A., Ghassemi A. (2016). A review of polymeric membranes and processes for potable water reuse. Prog. Polym. Sci..

[61] Dangeti S., McBeth J.M., Roshani B., Vyskocil J.M., Rindall B., Chang W. (2020). Microbial communities and biogenic Mn-oxides in an on-site biofiltration system for cold Fe-(II)- and Mn(II)-rich groundwater treatment. Sci. Total Environ..

[62] Mohammadmoradi P., Taheri S., Bryant S.L., Kantzas A. (2018). Solvent diffusion and dispersion in partially saturated porous media: An experimental and numerical pore-level study. Chem. Eng. Sci..

[63] Yang P., Moloney M.G., Zhang F., Ji W. (2018). Surface hydrophobic modification of polymers with fluorodiazomethanes. Mater. Lett..

[64] Khan S.A., Zulfiqar U., Hussain S.Z., Zaheer U., Hussain I., Husain S.W., Subhani T. (2017). Fabrication of superhydrophobic filter paper and foam for oil–water separation based on silica nanoparticles from sodium silicate. J. Sol-Gel Sci. Technol..

[65] de Júlio M.F., Ilharco L.M. (2014). Superhydrophobic hybrid aerogel powders from waterglass with distinctive applications. Microporous Mesoporous Mater..

[66] Yue C., Liu J., Zhang H., Dai L., Wei B., Chang Q. (2018). Increasing the hydrophobicity of filter medium particles for oily water treatment using coupling agents. Heliyon.

