# Supplementary material for: Congo Red Decolorization Using Textile Filters and Laccase-Based Nanocomposites in Continuous Flow Bioreactors
Source: Nanomaterials (Basel). 2020 Jun 24;10(6):1227. doi: 10.3390/nano10061227 (PMC7353132; doi:10.3390/nano10061227)
Supplement: Supplementary file 1 [file nanomaterials-10-01227-s001.pdf]

nanomaterials

Supplementary Materials

Congo Red Decolorization Using Textile Filters and Laccase-Based Nanocomposites in Continuous Flow Bioreactors

Natalia Lopez-Barbosa [1,2,†], Sergio Leonardo Florez [1,†], Juan C. Cruz [2], Nancy Ornelas-Soto [3] and Johann F. Osma [1,*]

[1] CMUA, Department of Electrical and Electronics Engineering, Universidad de los Andes, Cra. 1E No. 19a-40, Bogotá DC 111711, Colombia; n.lopez10@uniandes.edu.co (N.L.B.); sl.florez10@uniandes.edu.co (S.L.F.)
[2] Department of Biomedical Engineering, Universidad de los Andes, Cra. 1E No. 19a-40, Bogotá DC 111711, Colombia; jc.cruz@uniandes.edu.co
[3] Laboratorio de Nanotecnología Ambiental, Escuela de Ingeniería y Ciencias, Tecnológico de Monterrey, N. L., Monterrey 64849, México; ornel@tec.mx
* Correspondence: jf.osma43@uniandes.edu.co; Tel.: +57-1-339-4949
† Both authors contributed equally to this manuscript.

Supplementary tables

Table S1. Decolorization percentage and residual activity measurements of free laccase (Free), immobilized laccase on SiO_2 nanoparticles (IL), free laccase and NW (FreeNW), free laccase and NA (FreeNA), immobilized laccase on NW SiO_2 nanoparticles (ILNW), and immobilized laccase on NA SiO_2 nanoparticles (ILNA).

	Free		IL		Free-NW	
Time	% Decolorization	Residual activity [U/L]	% Decolorization	Residual activity [U/L]	% Decolorization	Residual activity [U/L]
5	78.53	0.00	70.02	40.07	77.32	0.00
10	67.72	0.00	46.27	40.07	74.50	83.48
15	61.33	100.17	35.69	40.07	70.11	273.80
20	61.33	156.93	30.02	76.80	67.73	273.80
25	38.82	160.27	22.54	123.54	67.57	310.53
30	38.82	317.21	22.54	220.37	64.14	310.53
35	26.38	317.21	19.18	323.88	63.58	310.53
40	26.38	500.85	19.18	323.88	58.20	310.53
45	26.38	651.11	17.52	340.58	49.76	387.32
50	21.05	651.11	17.52	494.17	49.76	387.32
55	21.05	651.11	17.30	631.07	39.61	430.73
60	16.44	734.58	17.30	681.16	34.39	430.73
65	16.44	788.00	17.30	681.16	34.39	430.73
70	9.85	788.00	13.65	681.16	34.39	430.73
75	9.85	788.00	13.65	727.90	29.14	520.88
80	9.85	808.04	13.65	727.90	29.14	520.88
85	9.85	848.11	13.65	727.90	29.14	520.88
90	8.07	848.11	13.65	727.90	29.14	520.88
	IL-NW		Free-NA		IL-NA	
Time	% Decolorization	Residual activity [U/L]	% Decolorization	Residual activity [U/L]	% Decolorization	Residual activity [U/L]
5	45.84	60.10	84.42	163.61	87.61	0.00
10	44.10	93.49	69.55	186.98	68.18	89.04

15	44.22	227.05	58.60	241.52	57.17	89.04
20	44.22	310.53	50.65	249.31	51.54	133.56
25	38.27	380.65	46.86	272.69	47.87	133.56
30	38.27	464.12	43.87	296.06	47.18	133.56
35	29.31	504.19	42.99	296.06	47.04	133.56
40	29.31	504.19	42.83	311.64	46.22	178.08
45	27.48	504.19	42.71	311.64	45.42	178.08
50	27.48	567.63	41.63	311.64	45.32	267.12
55	22.54	567.63	40.40	397.34	44.59	267.12
60	22.54	621.05	39.23	397.34	44.25	311.64
65	22.54	621.05	39.16	397.34	43.29	356.16
70	22.54	664.46	38.11	397.34	41.05	356.16
75	21.75	664.46	36.97	397.34	40.89	356.16
80	21.75	664.46	35.66	397.34	39.93	356.16
85	21.75	664.46	34.54	397.34	39.60	356.16
90	21.75	664.46	33.41	397.34	39.37	356.16

Supplementary Figures

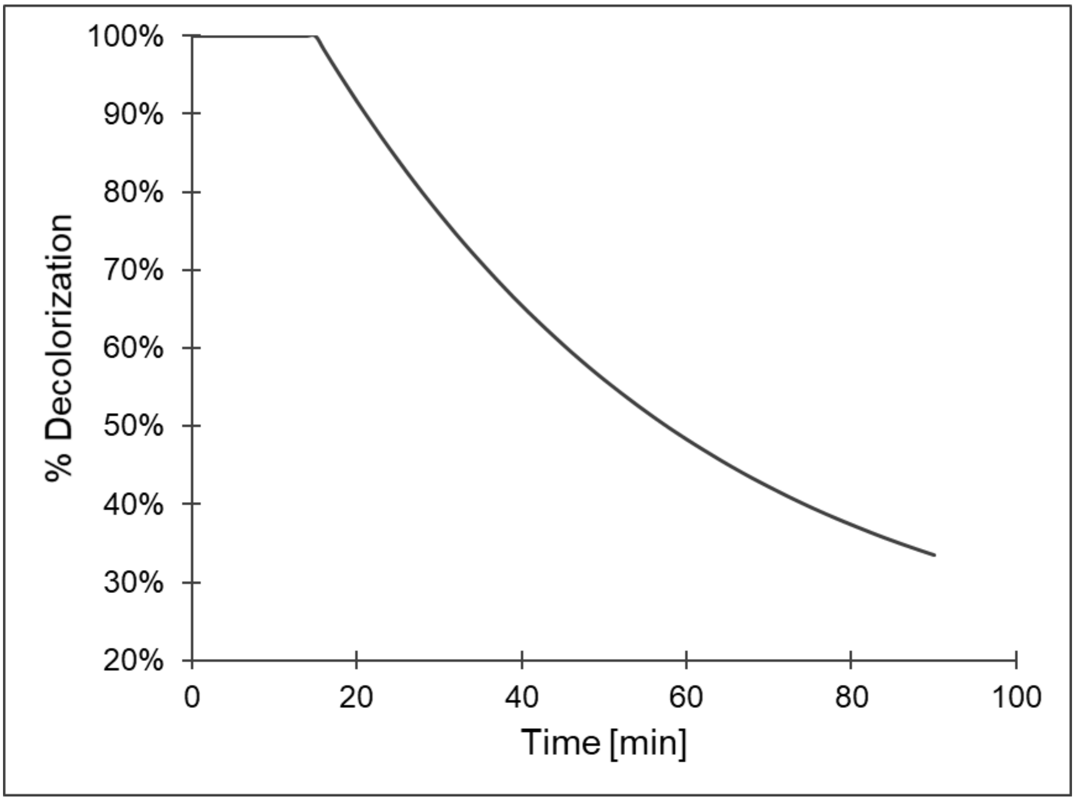

Figure S1. Simulation results of combining IL and ILNW filters.

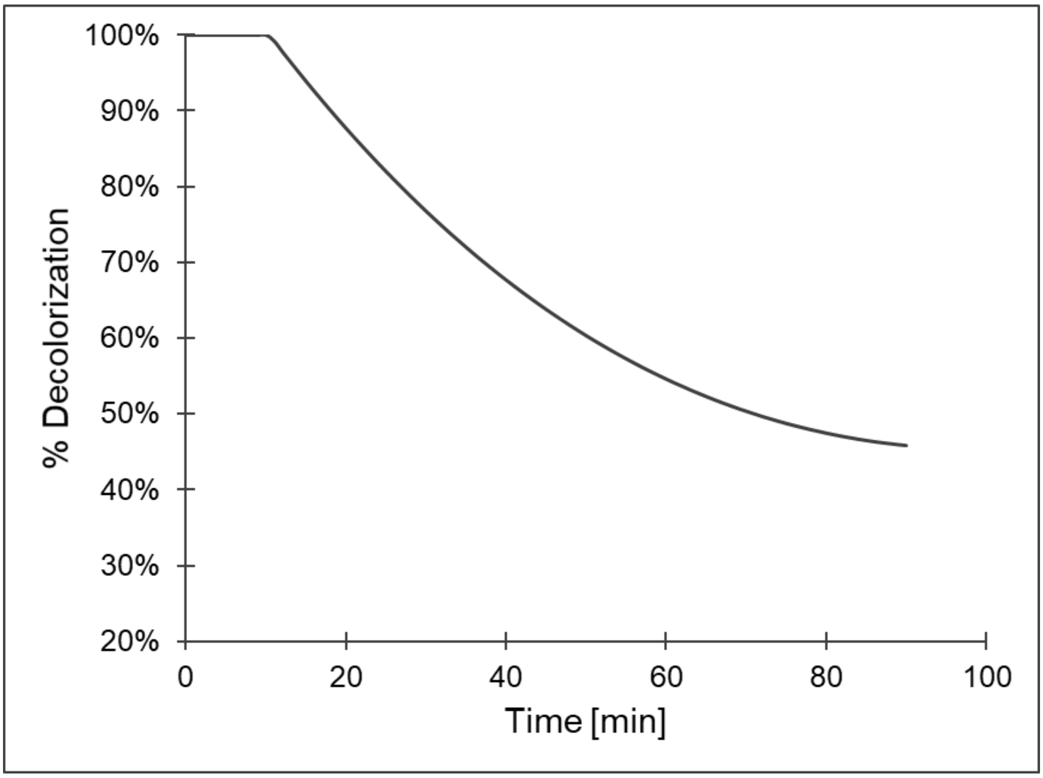

Figure S2. Simulation results of combining FreeNW and ILNW filters.

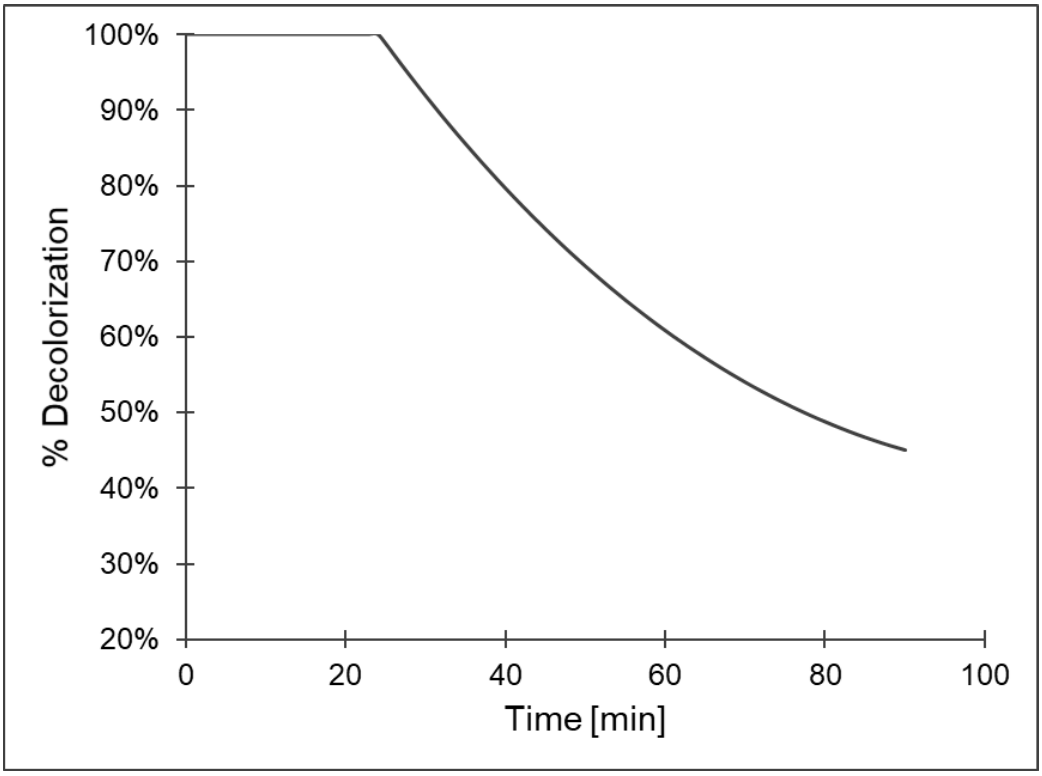

Figure S3. Simulation results of combining FreeNW and IL filters.

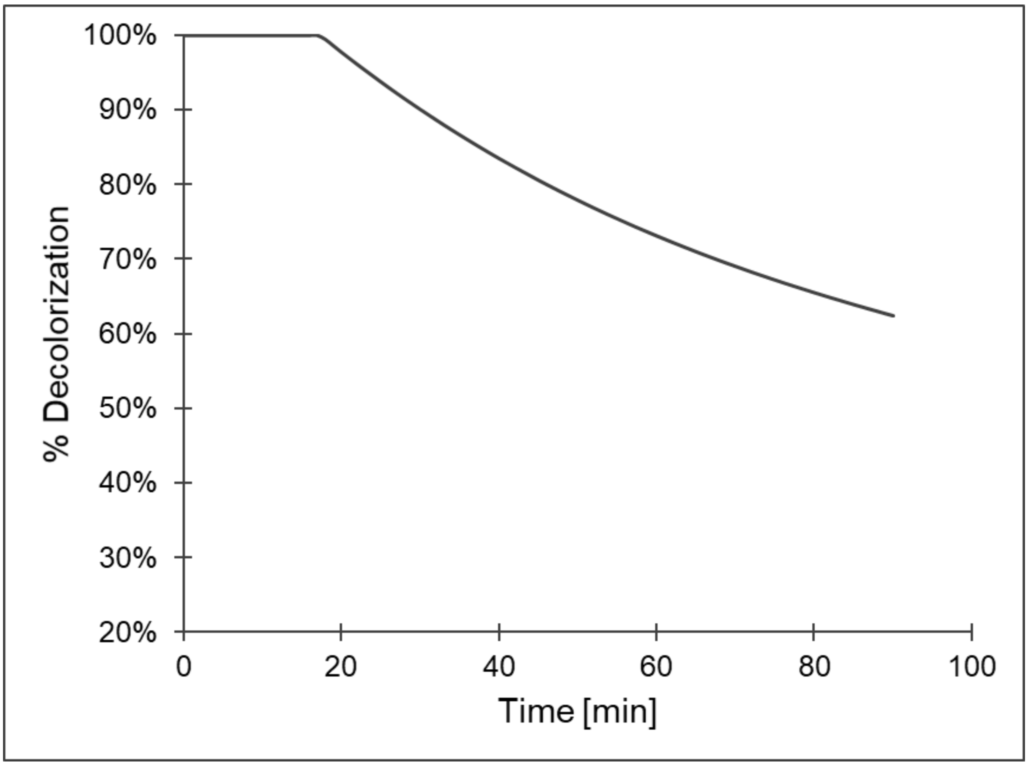

Figure S4. Simulation results of combining ILNW and ILNA filters.

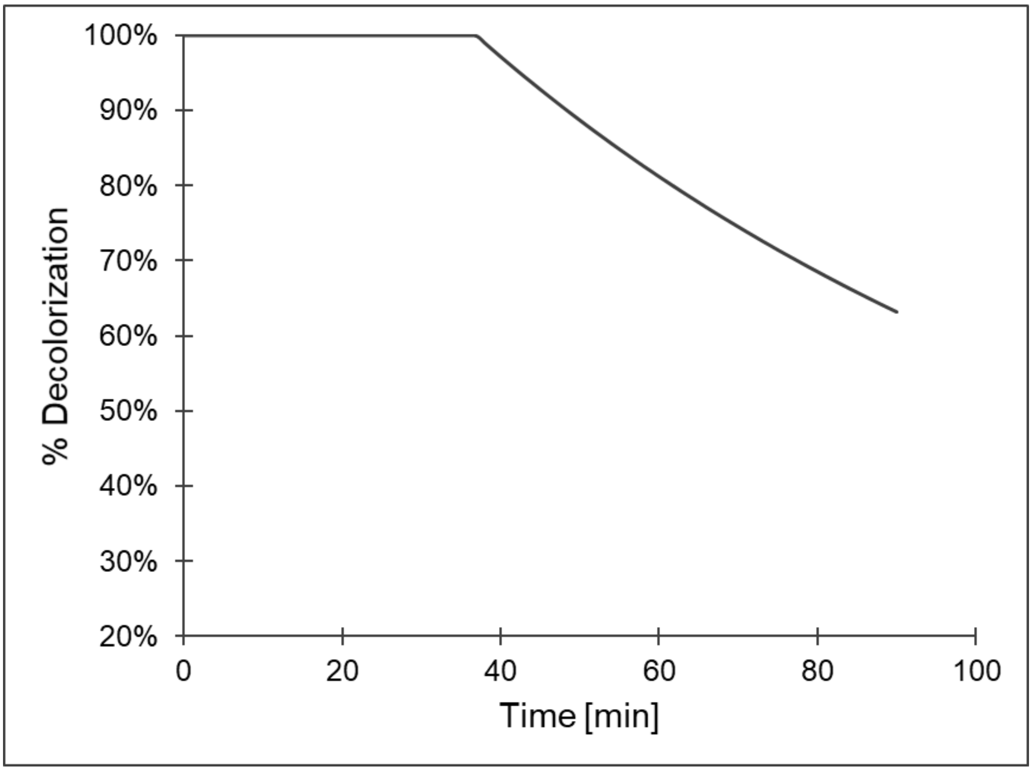

Figure S5. Simulation results of combining IL and ILNA filters.

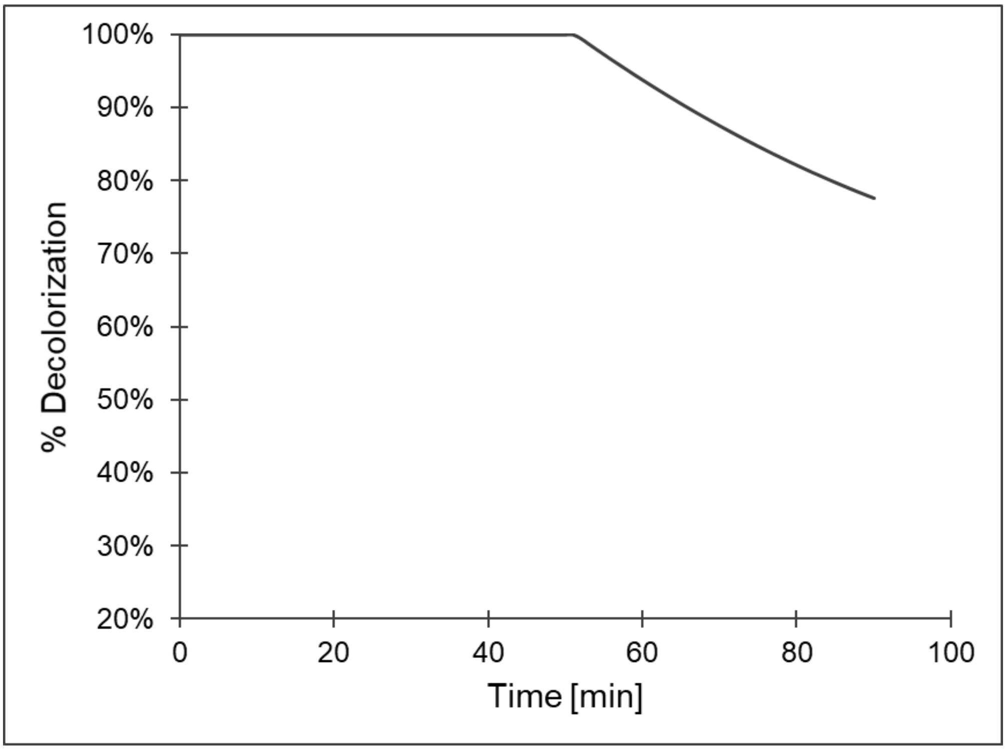

Figure S6. Simulation results of combining FreeNW and ILNA filters.

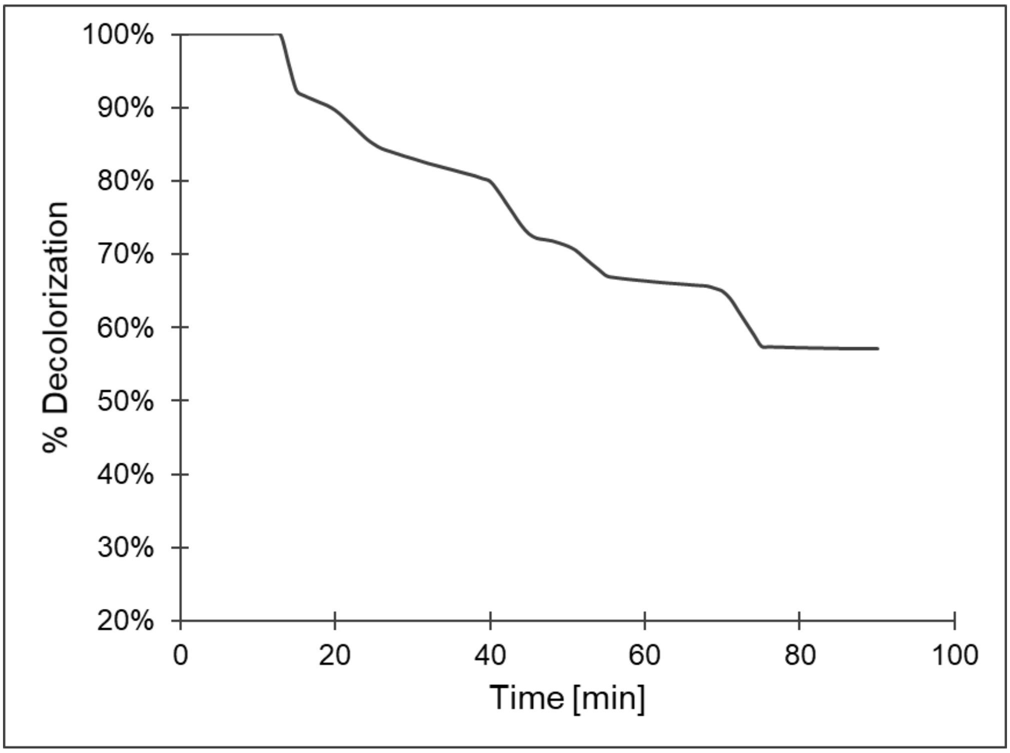

Figure S7. Simulation results of combining FreeNA and ILNW filters.

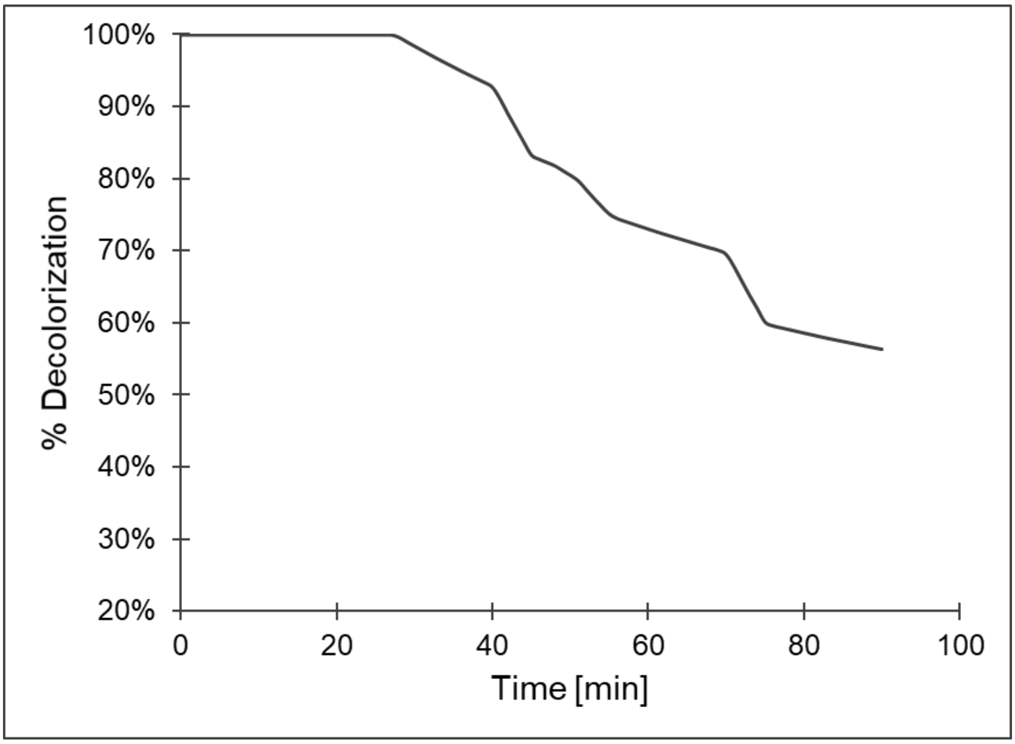

Figure S8. Simulation results of combining FreeNA and IL filters.

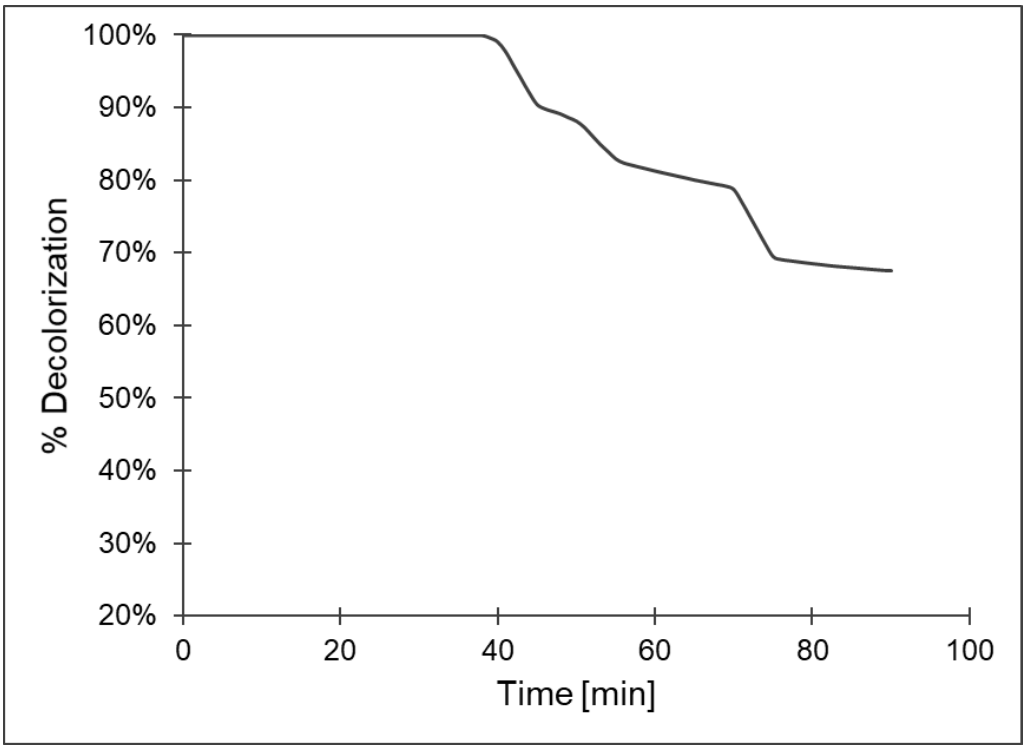

Figure S9. Simulation results of combining FreeNA and FreeNW filters.

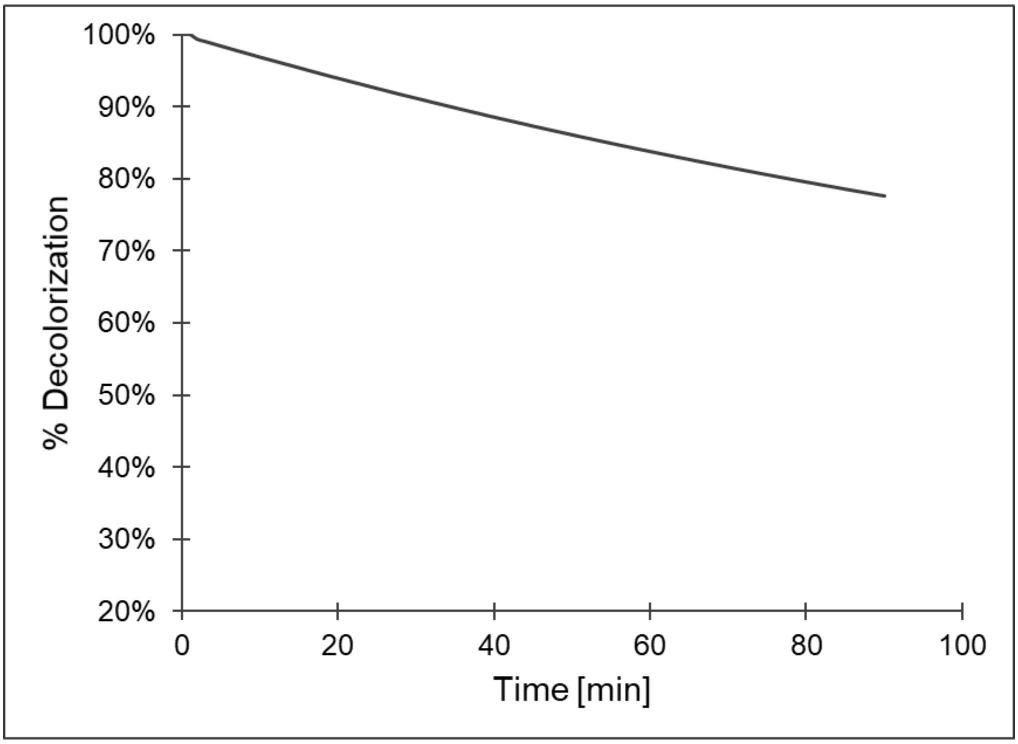

Figure S10. Simulation results of combining FreeNA and ILNA filters.

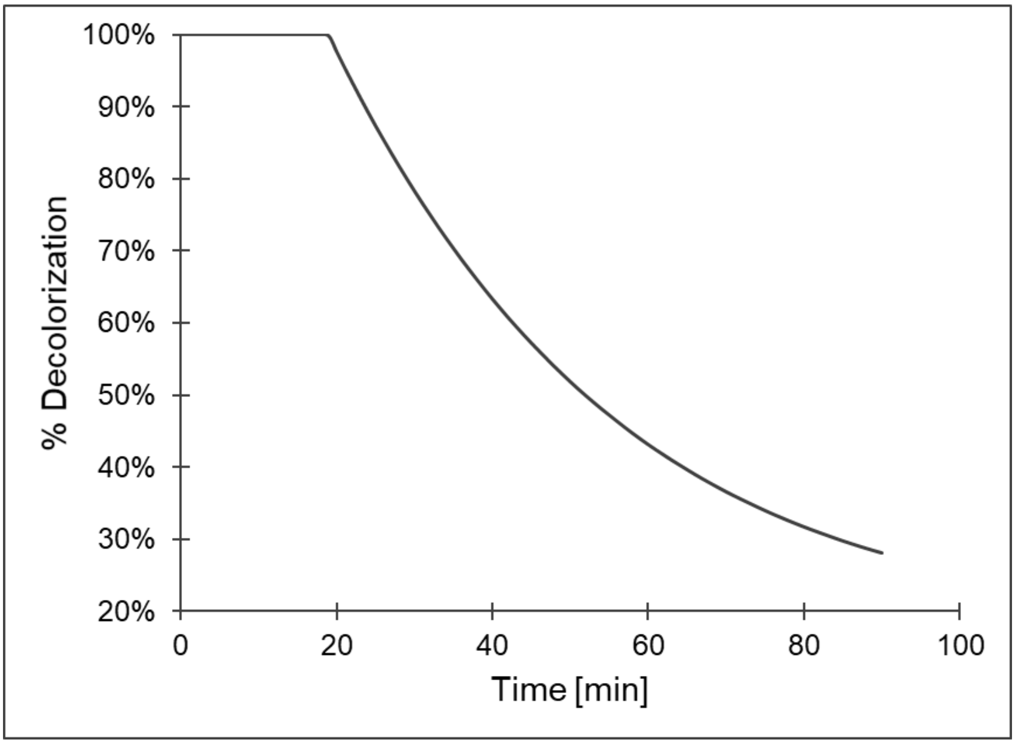

Figure S11. Simulation results of combining Free and ILNW filters.

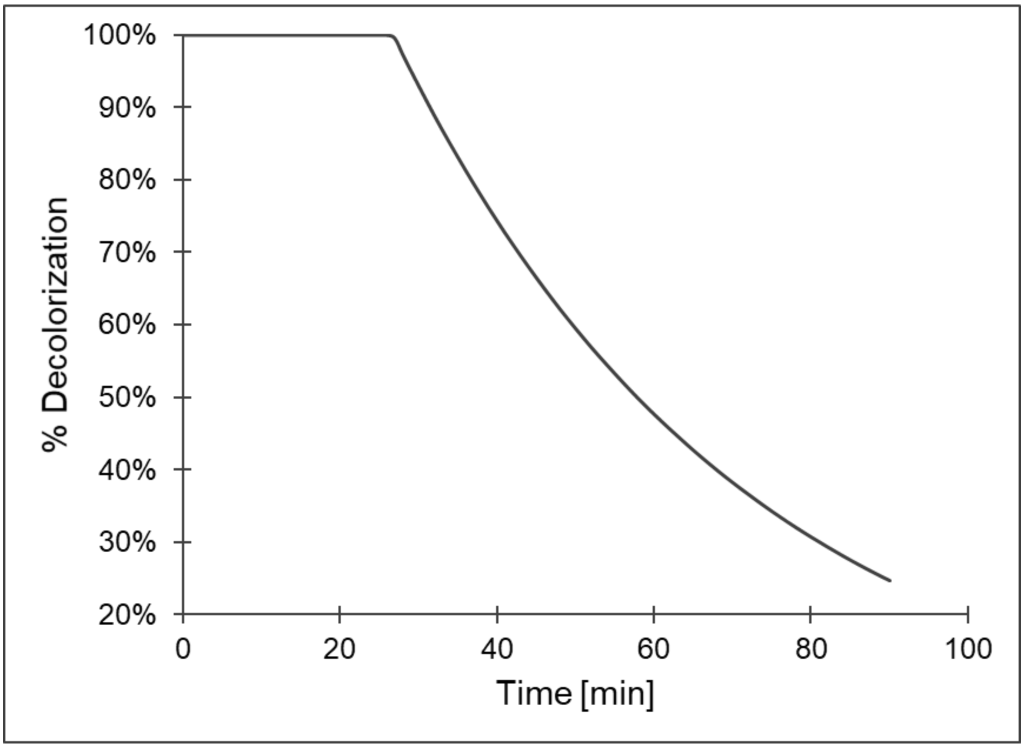

Figure S12. Simulation results of combining Free and IL filters.

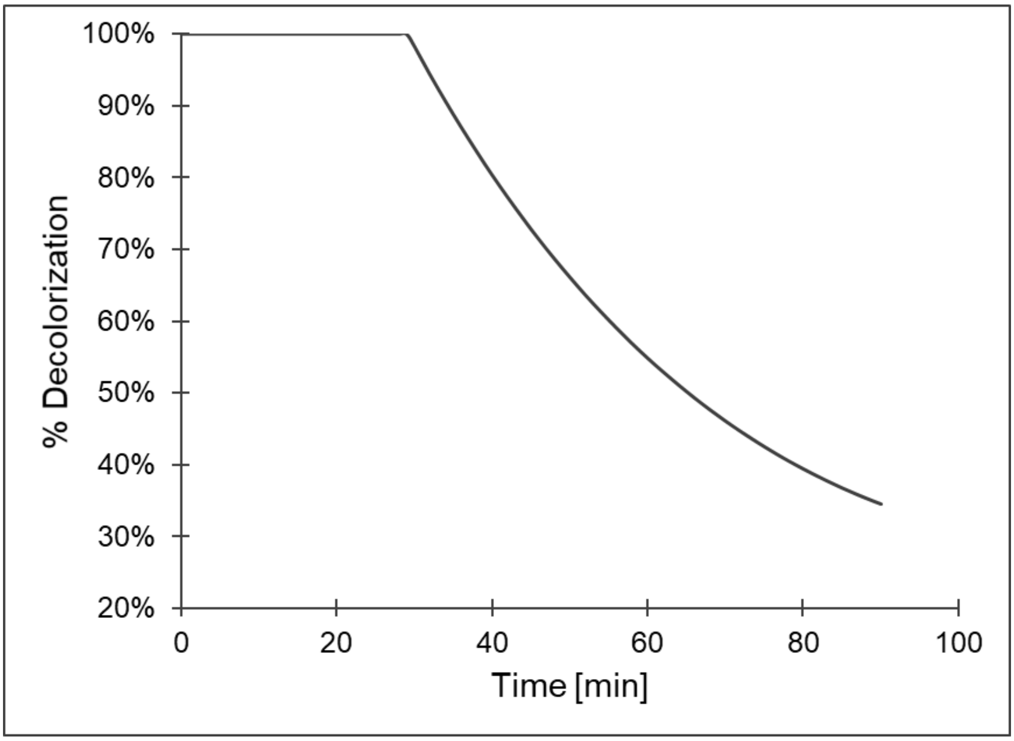

Figure S13. Simulation results of combining Free and FreeNW filters.

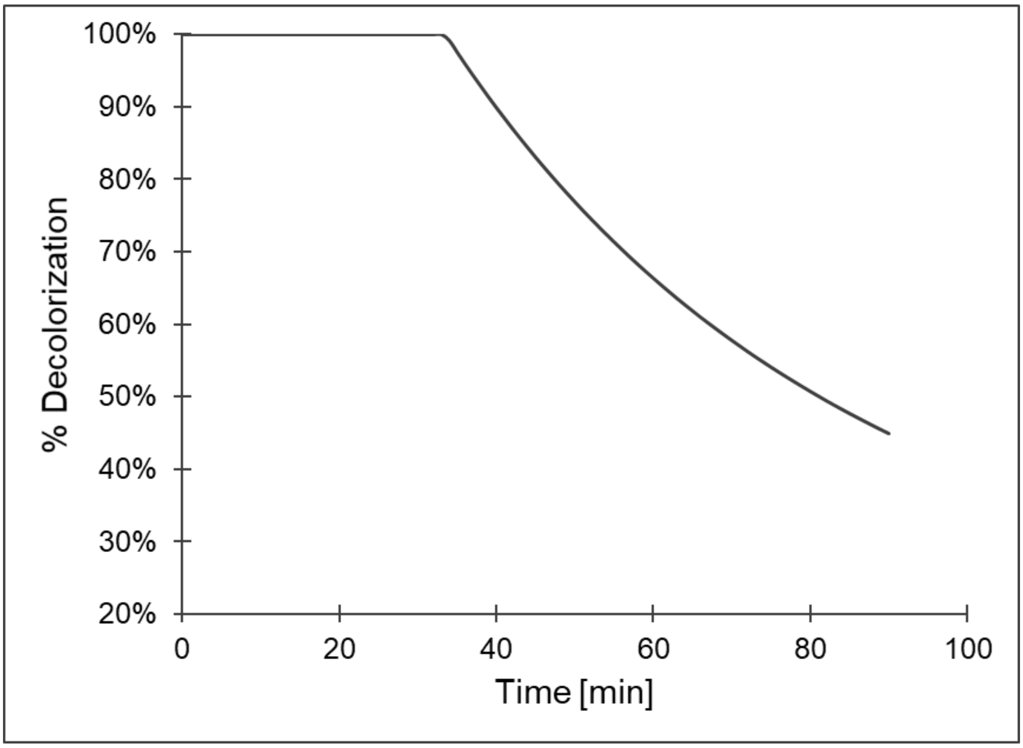

Figure S14. Simulation results of combining Free and ILNA filters.

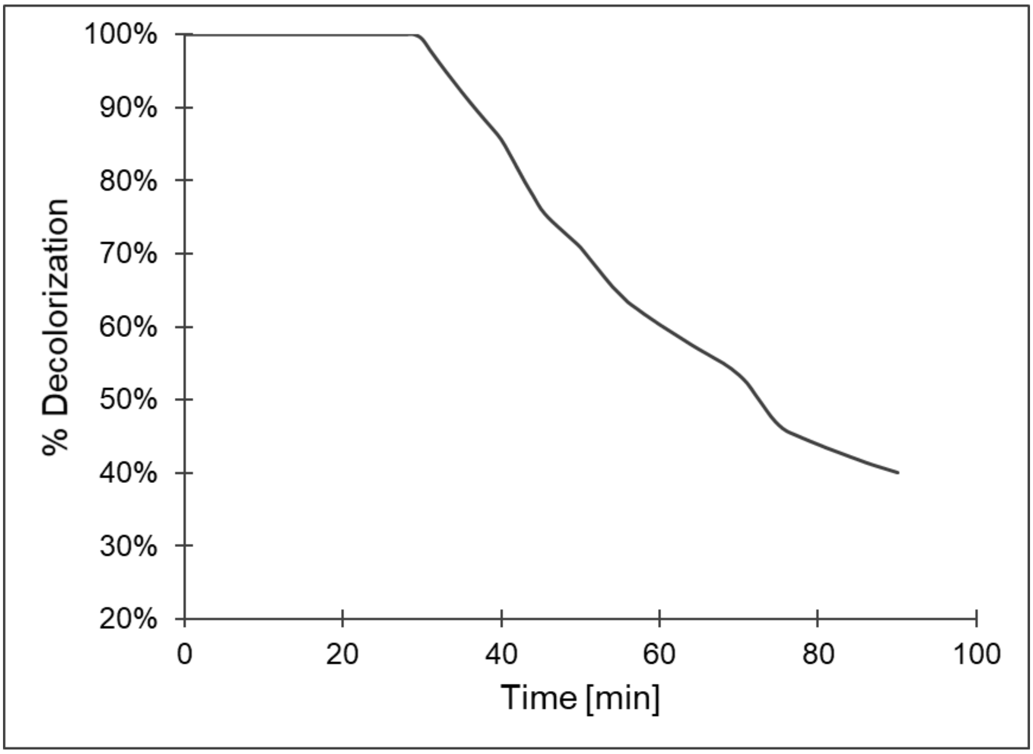

Figure S15. Simulation results of combining Free and FreeNA filters.

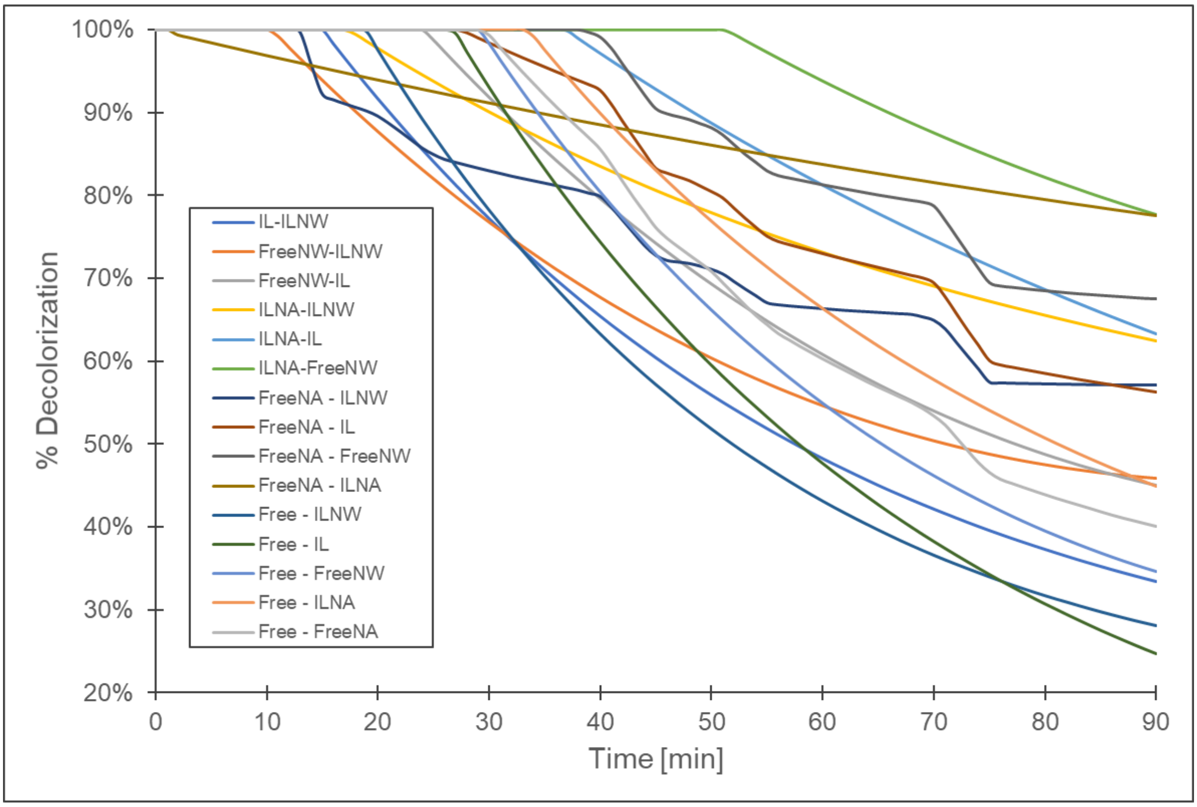

Figure S16. Simulation results of all two-combinations of the different types of filters.
